# Image Reconstruction Requirements for Short-Range Inductive Sensors Used in Single-Coil MIT

**DOI:** 10.3390/s24092704

**Published:** 2024-04-24

**Authors:** Joe R. Feldkamp

**Affiliations:** Tayos Corp., 1816 Gallagher Ln, Louisville, CO 80027, USA; jrfeld@tayoscorp.com

**Keywords:** electrical conductivity, magnetic induction tomography, short range sensor, single-coil scanning, inductive loss, Fredholm integral, inverse problem

## Abstract

MIT (magnetic induction tomography) image reconstruction from data acquired with a single, small inductive sensor has unique requirements not found in other imaging modalities. During the course of scanning over a target, measured inductive loss decreases rapidly with distance from the target boundary. Since inductive loss exists even at infinite separation due to losses internal to the sensor, all other measurements made in the vicinity of the target require subtraction of the infinite-separation loss. This is accomplished naturally by treating infinite-separation loss as an unknown. Furthermore, since contributions to inductive loss decline with greater depth into a conductive target, regularization penalties must be decreased with depth. A pair of squared L2 penalty norms are combined to form a 2-term Sobolev norm, including a zero-order penalty that penalizes solution departures from a default solution and a first-order penalty that promotes smoothness. While constraining the solution to be non-negative and bounded from above, the algorithm is used to perform image reconstruction on scan data obtained over a 4.3 cm thick phantom consisting of bone-like features embedded in agarose gel, with the latter having a nominal conductivity of 1.4 S/m.

## 1. Introduction

Magnetic induction tomography (MIT), as applied to the determination of an electrical conductivity distribution within low-conductivity biological targets, is ordinarily accomplished with a system consisting of two or more coils, each commonly using circular loop geometry [1]. Choice of coil diameter largely depends upon the intended application, with larger diameters providing improved sensitivity to deeply buried features, while smaller diameter coils offer improved resolution for shallow features. Typically, coil diameter ranges from ∼5 cm up to ∼40 cm, but no larger than nominal target dimensions [1,2,3]. Achieving both interior sensitivity and adequate resolution remains a challenge according to Klein et al. [2], which considers both circular and noncircular coil geometry.

An alternative approach to multicoil MIT, as recently demonstrated [4], uses a single, multiloop type coil to provide both excitation and sensing. Image reconstruction in this case is made possible by an analytic formula that connects a 3D electrical conductivity distribution with measured inductive loss via a Fredholm integral equation of the first kind. Primary limitations of the Fredholm integral include a restriction to short circular loop coil geometry, electrical conductivities beneath ∼200 S/m, and targets having near-uniform relative permittivity and permeability. Though uniform relative permittivity is usually not present, even in phantoms, the conductivity limitation is certainly acceptable for biological materials where conductivity is typically less than ∼2.0 S/m [5,6,7,8]. Thus far, single-coil MIT scans have been demonstrated only for coil diameters less than or equal to ∼5 cm, where interest has focused on shallow features, such as the lumbar spine and near-surface pressure ulcers.

An important distinction between multicoil and single-coil MIT electronics lies in the manner of excitation and signal detection. While multicoil MIT methods rely upon detection of the phase difference between excitation and response fields [9], the single-coil MIT system discussed herein relies upon inductive loss detection in an RLC tank circuit. Either strategy encounters increasingly serious problems as electrical conductivity becomes small—either the induced field becomes too small to detect a phase difference, or inductive loss becomes small when compared with background noise. An advantage of the inductive loss measurement is that loss depends linearly upon conductivity as long as the Fredholm integral remains valid.

Instrument sensitivity to interior features declines with depth into a target for either single- or multicoil MIT [2,10,11]. With single-coil MIT, this is well visualized from the Fredholm integral where the kernel may decline by more than 10-fold at a distance of one coil radius away from the coil plane, though actual decay is dependent upon radial location. Because of declining sensitivity with depth, care must be taken in methods used for regularization when attempting to invert the ill-conditioned Fredholm integral. Choice of penalty types, with built-in depth-dependence, can determine whether or not image reconstruction is able to resolve both near-surface and interior features.

Data collected from single-coil MIT scans have thus far been processed with Tikhonov-regularized image reconstruction methods involving a single L2 type penalty term. These have either suppressed rapid spatial variations in electrical conductivity through the solution gradient norm, or departures in electrical conductivity from a precomputed default solution. This work combines both into a single depth-dependent penalty, more generally regarded as a 2-term Sobolev norm.

Regardless of regularization choices implemented during image reconstruction, several other features are indispensible for successful single-coil MIT. Firstly, we must recognize that inductive sensor response diminishes quickly with sensor–target distance. Since all inductive sensor measurements must be made relative to sensor response at infinite separation between target and sensor coil, it is essential to locate this asymptote. Here, we illustrate an image reconstruction method that treats asymptote location as an unknown, alongside conductivity, so that all other measurements properly reflect target–coil interaction relative to infinite separation.

Secondly, since no induction coil is ideal, data preprocessing should remove tank circuit losses associated with coil–target parasitic capacitance. Though usually small, this can make a difference in image quality, as recently shown [12]. Current work uses a Texas Instruments LDC1101 chip to measure tank circuit losses for an RLC resonant circuit that includes the sensor coil [13,14], which allows a distinction between inductive and capacitive losses.

Finally, image reconstruction for single-coil MIT should use any available a priori information about the target, such as an expected range for conductivity. For biological samples, electrical conductivity can be reliably expected to fall into a range from 0.0 to 2.0 S/m. Thus, the methods reported here use Lagrange multiplier methods to constrain the solution between known bounds. Any other a priori information should also be used, such as the known spatial boundaries of the target. Finite element methods used here leverage that information.

The next section provides details of the image reconstruction method used for single-coil MIT that incorporates the features discussed above. Since the goal is to showcase these features for a relatively small coil, they are illustrated in the context of quadratically regularized least squares, which is likely known to the reader. Following the discussion of the algorithm, image reconstruction is performed on experimental data collected from a single-coil scan over a phantom using a 3D robotic gantry. Phantom construction involves the placement of “bone-like” inclusions throughout an agarose matrix of dimensions 30 × 30 × 4.5 cm—the last dimension giving specimen depth, which is about twice the coil radius chosen for scanning in this work.

## 2. Single-Coil MIT Image Reconstruction

### 2.1. Dual-Penalty Regularized Least Squares

Image reconstruction of data obtained from a single-coil scan is based upon an analytical formula linking 3D electrical conductivity distribution $\sigma_{c}$ within a target to predicted inductive loss in the sensor coil [4]. This formula fully accounts for skin effects provided that conductivity is much smaller than ∼$\sqrt{2\pi }/\left(\mu \nu a^{2}\right)$ (μ is magnetic permeability, ν is frequency, and *a* is coil radius).

(1) $$Z=\frac{\mu^{2}\omega^{2}}{4\pi^{2}}\sum_{j,k}\sqrt{\rho_{j}\rho_{k}}\int d^{3}x_{c}\frac{\sigma_{c}\left({\vec{r}}_{c}\right)}{\rho }Q_{1/2}\left(\eta_{j}\right)Q_{1/2}\left(\eta_{k}\right)$$

Integration in Equation (1) is entirely in the coil frame, with origin located at the coil center and XY plane coplanar with the coil plane—a “*c*” subscript indicates the coil frame. The argument of toroid function $Q_{1/2}$ is given by

(2) $$\eta_{j}=\frac{\rho^{2}+\rho_{j}^{2}+z_{c}^{2}}{2\rho \rho_{j}}\;\;\;;\;\;\;\;\rho^{2}=x_{c}^{2}+y_{c}^{2}$$

Radial distance ρ is subscripted when locating coil loop *j* or *k*. Equation (1) has shown very close agreement with experiment in several studies [13], and forms the basis for image reconstruction from single-coil scan data. It predicts the expected inductive contribution to loss in a tank circuit, which is dependent on the position and orientation of the sensor coil. To make this clearer, Equation (1) can be written as a convolution integral relating coil position $\vec{c}$ and rotation matrix $\tilde{\Omega }$ to measured loss [4]:

(3) $$Z\left(\vec{c}\right)\;\;\;=\;\;\int \sigma_{l}\left(\vec{r}\right)\;K\left({\tilde{\Omega }}^{T}\left(\vec{r}-\vec{c}\right)\right)dxdydz$$

The integration in Equation (3) is now fully from the perspective of the lab frame, with a subscript “*l*” attached to conductivity as a reminder. ${\tilde{\Omega }}^{T}$ rotates a vector in the lab frame back to the coil frame, while vector $\vec{c}$ is from lab origin to coil center and $\vec{r}$ locates a field point in the lab frame. Because of cylindrical symmetry about the coil axis, $\tilde{\Omega }$ is the identity matrix if coil and XY planes are parallel. Parallel configurations are common with many scan methods.

The kernel $K\left({\vec{r}}_{c}\right)$ in Equation (3) is given as the sum over the loop radii of the set of very short concentric solenoids connected in series:

(4) $$K\left({\vec{r}}_{c}\right)=\frac{\mu^{2}\omega^{2}}{4\pi^{2}}\frac{1}{\rho }\sum_{j,k}\sqrt{\rho_{j}\rho_{k}}Q_{1/2}\left(\eta_{j}\right)Q_{1/2}\left(\eta_{k}\right)$$

Kernel $K\left({\vec{r}}_{c}\right)$ directly assesses sampling and assigns the extent of importance given to each location within a target—it is zero along the coil axis. Example kernel plots have been shown elsewhere [4], which demonstrate the loss of sensitivity to regions farther from the coil. After inductive loss is repeatedly measured in the vicinity of a conductive target, the measurement set is used to solve for conductivity by inversion of Equation (1) or Equation (3).

Image reconstruction begins by discretizing the integral of Equation (3). Here, electrical conductivity is expressed as a linear combination of basis functions over a finite element mesh consisting of *N* nodes, that spans just the target:

(5) $$\sigma_{l}\left(\vec{r}\right)=\sum_{j=1}^{N}\sigma_{j}\varphi_{j}\left(\vec{r}\right)$$

After introducing this expansion into Equation (3), we have a loss prediction for each coil location during a scan:

(6) $$Z\left({\vec{c}}_{i}\right)\;\;\;=\;\;\sum_{j=1}^{N}\sigma_{j}\int \varphi_{j}\left(\vec{r}\right)K\left({\tilde{\Omega }}^{T}\left(\vec{r}-{\vec{c}}_{i}\right)\right)dxdydz=\tilde{T}\vec{\sigma }$$

The remaining integral is over known functions and is evaluated for each element to build model matrix $\tilde{T}$. The toroid function is evaluated here using the algorithm of Fettis [15], which is especially effective in the difficult argument range between 1.0 and ∼1.1.

Inductive loss data, together with Equation (6), can be written as a matrix equation, with predicted loss $Z({\vec{c}}_{i})$ providing an approximation of the measured loss “$\vec{b}$”:

(7) $$\tilde{T}\vec{\sigma }\approx \vec{b}\;\;\;;\;\;\;\;\;\;T_{ij}=\int \varphi_{j}\left(\vec{r}\right)K\left({\tilde{\Omega }}^{T}\left(\vec{r}-{\vec{c}}_{i}\right)\right)dxdydz$$

Ordinarily, the number of unknowns contained in the vector $\vec{\sigma }$ exceeds the number of available equations, determined by the number of measurements. Thus, the system of Equation (7) is underdetermined. If measurements are nearly redundant, then the number of meaningful equations becomes smaller.

Even with good sampling, strategies are still needed to reduce the size of the solution space. Given that electrical conductivity is strictly positive, solution non-negativity is imposed. Another requirement that further shrinks the solution space is to enforce an upper conductivity bound. For example, the electrical conductivity in biological tissues is known to be less than ∼2.0 S/m [5,6,7,8]. With phantoms, the upper bound may be precisely known. Here, Lagrange multipliers and “active set” methods are used to keep the solution between zero and some upper bound.

Though bound constraints are imposed, there still is no unique solution for Equation (7). Thus, Tikhonov regularization is employed by minimizing the sum of the error norm and two weighted L2 norms, further limiting the solution space. Multipenalty regularization has previously been used in 1D applications, yielding results superior to single-penalty regularization schemes [16]. Here, one L2 norm penalizes departures from an overall conductivity average (the default solution—see Equation (12) of [4]) and a second penalizes solutions with large spatial gradients:

(8) $$\min\;\;\frac{1}{2}{∥\tilde{T}\vec{\sigma }-\vec{b}∥}^{2}+\frac{1}{2}\tau^{2}{∥\tilde{G}\;\vec{\sigma }∥}^{2}+\frac{1}{2}\alpha^{2}{∥\tilde{D}\left(\vec{\sigma }-\vec{\beta }\right)∥}^{2}\;$$

Anticipating a finite element mesh with conductivity unknown at *N* nodes, minimization problem (8) is subjected to the constraints:

(9) $$s.t.\;\;\;\;0\leq \sigma_{j}\leq \sigma_{\max}\;\;\;\left(j=1,2,\ldots ,N\right)$$

Matrix $\tilde{G}$ is the conductivity gradient matrix, consisting of a first-order differential operator, which has a structure dependent upon the type of finite element mesh and associated basis functions. This first-order penalty norm remains unchanged if a constant is added to the unknown conductivity. Thus, the objective function in (8) may be written as

(10) $$\frac{1}{2}{∥\tilde{T}\vec{\sigma }-\vec{b}∥}^{2}+\frac{\tau^{2}}{2}{∥\tilde{G}\;\left(\vec{\sigma }-\vec{\beta }\right)∥}^{2}+\frac{\alpha^{2}}{2}{∥\tilde{D}\left(\vec{\sigma }-\vec{\beta }\right)∥}^{2}$$

The penalty term involving diagonal matrix $\tilde{D}$ penalizes solutions that deviate from the default solution, $\vec{\beta }$. Inasmuch as $\tilde{D}$ applies a scalar to the conductivity displacement, this penalty is a zero-order contribution to the overall penalty. Higher-order penalty terms could be developed and summed together, but are not considered here.

Through either $\tilde{D}$ or $\tilde{G}$, differing penalties may be imposed on specific components of the conductivity vector or gradient. For example, solution components at deeper target locations may be less penalized. Given the inherently weaker coil EM field at increased depth, a reduced penalty is usually necessary to improve solution sensitivity to interior regions.

The two penalty terms may now be combined into a single L2 normed penalty. First, the quantity to minimize, from expression (10), is rewritten as

(11) $$\frac{1}{2}{∥\tilde{T}\vec{\sigma }-\vec{b}∥}^{2}+\frac{\tau^{2}}{2}{∥\tilde{G}\;\left(\vec{\sigma }-\vec{\beta }\right)∥}^{2}+\frac{\tau^{2}}{2}{∥\frac{\alpha }{\tau }\tilde{D}\left(\vec{\sigma }-\vec{\beta }\right)∥}^{2}\;$$

This allows the two penalty terms to be combined as

(12) $$\frac{1}{2}{∥\tilde{T}\vec{\sigma }-\vec{b}∥}^{2}+\frac{\tau^{2}}{2}{∥\tilde{H}\;\left(\vec{\sigma }-\vec{\beta }\right)∥}^{2}\;\;\;;\;\;\;\;\tilde{H}\equiv \left[\begin{array}{c} \tilde{G}\\ \frac{\alpha }{\tau }\tilde{D} \end{array}\right]$$

The new matrix $\tilde{H}$ has an increased number of rows compared to either $\tilde{G}$ or $\tilde{D}$. Letting *E* represent the number of elements in a 3D mesh, the number of rows in $\tilde{G}$ equals 3 ×E, while $\tilde{D}$ has *N* rows. To more clearly see the structure of matrix $\tilde{G}$, we apply the gradient operator to the conductivity in Equation (5) to yield for element *e*:

(13) $$\nabla_{e}\sigma =\sum_{j}^{N}\sigma_{j}\nabla \varphi_{j}$$

Equation (13) can be written in matrix form:

(14) ∇eσ→·∂σ∂x|e∂σ∂y|e∂σ∂z|e·=G˜σ→ ; e=1,2,…,E

Entries within $\tilde{G}$ consist of the vectors $\nabla \varphi_{j}$, obtained from the basis functions, with the matrix entry location determined by the element and node-numbering scheme used. Any particular element within the matrix $\tilde{G}$ can be written as

(15) $${\tilde{G}}_{ij}=\frac{\partial \varphi_{j(k,e)}}{\partial x_{m}}$$

The size of $\tilde{G}$ is 3E×N, while indices *i*, *k*, and *m* are given by

(16) i=3(e−1)+m;k=1,2,…,l(e);m=1,2,3

Element number is given by *e*, while m=1,2,3 correspond to x,y,z Cartesian coordinates. Index *j* is determined by the way the mesh elements and nodes are numbered. Thus, j(k,e) is determined according to how the particular local node number *k*, attached to element *e*, maps to a global node number *j*. The number of nodes attached to an individual element *e* is l(e). Prismatic elements are chosen here.

A simple approach to verifying the structure of matrix (12) in coding is to apply the matrix to a trial conductivity vector for a virtual target with a prescribed linear variation in conductivity across the mesh. The result should equal the assigned slope in each of the *X*, *Y*, and *Z* directions. If this procedure is used when a global constant (or element-wise constant) is added to the trial function, the results of Equation (11) are unchanged, so only solutions containing spatial changes in conductivity are penalized via matrix $\tilde{G}$.

Commonly, $\tilde{G}$ has more rows than columns, so that its rank might be expected to equal *N*. But because Equation (15) is unaffected by the addition of a constant to conductivity, $\tilde{G}$ is column rank deficient—specifically, column rank equals N−1. To restore the column rank of $\tilde{G}$ to *N*, an additional, independent row vector could be appended to $\tilde{G}$. For example, all entries of this last row could be set equal to zero, except for entry *N*, which could be set exactly to 1.0. The consequence of this choice is that solutions exhibiting high conductivity at this particular node are penalized. Alternatively, working with both penalty types simultaneously via the matrix $\tilde{H}$ achieves the same effect so that the column rank of matrix $\tilde{H}$ has the desired value of *N*. Thus, $\tilde{G}$ requires no modification.

Both matrices $\tilde{T}$ and $\tilde{H}$ must be further modified to accommodate an “instrument offset”, a feature that is particularly unique to single-coil, scanning MIT. This is due to the rapid decay of inductive loss as the coil sensor is moved farther from the target, gradually approaching some asymptotic value. Rather than try to specify or measure this asymptotic value, which is difficult, it is treated as an unknown. Therefore, the vector of unknowns is modified to

(17) $${\vec{\sigma }}_{o}=\left(b_{\infty },\;\;\vec{\sigma }\right)$$

The first entry is the instrument offset, or sensor reading asymptotically approached at infinite distance. To accommodate the offset in minimization problem (12), a new first column of $1^{\prime }$ s must be added to $\tilde{T}$, producing ${\tilde{T}}_{0}$, while $\tilde{H}$ is modified to include a new first row and first column, consisting of $0^{\prime }$ s, except for the (1,1) entry which is identically set equal to 1.0. The balance of the new ${\tilde{H}}_{0}$ is the previously assigned $\tilde{H}$, which has full column rank *N*. Since all sensor readings need to be relative to the asymptotic sensor reading, determination of the sensor asymptote is necessary, even if small.

Matrix $\tilde{H}$ is further processed via QR decomposition. This leads to modification of the last term in problem (12):

(18) $$\begin{array}{c} {∥\tilde{H}\left(\vec{\sigma }-\vec{\beta }\right)\;∥}^{2}\to {∥{\tilde{H}}_{o}\;\left({\vec{\sigma }}_{o}-{\vec{\beta }}_{o}\right)∥}^{2}=\\ b_{\infty }^{2}+{∥\tilde{H}\;\left(\vec{\sigma }-\vec{\beta }\right)∥}^{2}=b_{\infty }^{2}+{∥\tilde{Q}\tilde{R}\;\left(\vec{\sigma }-\vec{\beta }\right)∥}^{2}=\\ b_{\infty }^{2}+{∥\tilde{R}\;\left(\vec{\sigma }-\vec{\beta }\right)∥}^{2}={∥{\tilde{R}}_{o}\;\left({\vec{\sigma }}_{o}-{\vec{\beta }}_{o}\right)∥}^{2} \end{array}$$

Result (18) follows since matrix $\tilde{Q}$ is orthonormal, while vector ${\vec{\beta }}_{0}$ has zero as its first entry. Therefore, minimization problem (12) is now written as

(19) $$\begin{array}{c} \min\;\;\frac{1}{2}{∥{\tilde{T}}_{o}{\vec{\sigma }}_{o}-\vec{b}∥}^{2}+\frac{1}{2}\tau^{2}{∥{\tilde{R}}_{o}\;\left({\vec{\sigma }}_{o}-{\vec{\beta }}_{o}\right)∥}^{2}\;\;\;\;\;\;\\ \;s.t.\;\;\;\;\sigma_{j}\geq 0\;\;\;\left(j=1,2,\ldots ,N+1\right) \end{array}$$

The subscript “0” is present to indicate that the problem now includes the unknown offset, or asymptote, which may also be constrained to be non-negative. Thus, ${\tilde{R}}_{0}$ has the same row and column modifications as ${\tilde{H}}_{0}$, while $\tilde{R}$ is unchanged.

### 2.2. Conductivity Constraints

Further progress requires that bounds are imposed on the solution, which is accomplished through building an objective function from (19) that adds in Lagrange multiplier terms:

(20) $$\frac{1}{2}{∥{\tilde{T}}_{0}{\vec{\sigma }}_{0}-\vec{b}∥}^{2}+\frac{1}{2}\tau^{2}{∥{\tilde{R}}_{o}\;\left({\vec{\sigma }}_{0}-{\vec{\beta }}_{0}\right)∥}^{2}\;\;+\sum_{k=1}^{K}\lambda_{k}\sigma_{k}^{a}$$

The constrained set of *K* unknowns {$\sigma_{k}^{a}$} is called the active set, denoted by superscript *a*, and is initially found by minimizing without constraints and noting which unknowns are out of bounds. If a multiplier associated with a lower (upper) bound is found to be negative (positive) in subsequent iterations, the corresponding unknown remains in the active set; otherwise, it is released. During any iteration, if an unknown is found to be out of bounds, it is then added to the active set in a subsequent iteration. This strategy pertains to conductivity unknowns and optionally the unknown “instrument offset”. Note that this algorithm simultaneously manages all constraints in any iterative step, rather than the one-at-a-time approach used elsewhere [17]. Iteration ceases when the active set no longer changes its membership, accomplished in fewer than ∼9 iterations for results reported here.

Before putting (20) into standard form, the displacement in conductivity relative to its target average, or default solution, is used rather than the conductivity itself:

(21) $${\vec{\chi }}_{0}={\vec{\sigma }}_{0}-{\vec{\beta }}_{0}$$

The first component of ${\vec{\chi }}_{0}$ is the instrument offset, since the first component of ${\vec{\beta }}_{0}$ is zero. Minimization problem (20) becomes

(22) $$\frac{1}{2}{∥{\tilde{T}}_{0}{\vec{\chi }}_{0}-{\vec{\delta }}_{l}∥}^{2}+\frac{1}{2}\tau^{2}{∥{\tilde{R}}_{0}\;{\vec{\chi }}_{0}∥}^{2}\;\;+\sum_{k=1}^{K}\lambda_{k}\sigma_{k}^{a}$$

The displacement in measured loss is given as

(23) $${\vec{\delta }}_{l}\equiv \vec{b}-{\tilde{T}}_{0}{\vec{\beta }}_{0}$$

Just as ${\vec{\chi }}_{0}$ is the displacement in conductivity, ${\vec{\delta }}_{l}$ is the associated displacement in the measured inductive loss, relative to the loss that would be expected if all material had a uniform conductivity given from ${\vec{\beta }}_{0}$.

Minimization problem (22) now has the new bounding constraints:

(24) $$0\leq {\vec{\chi }}_{o}+{\vec{\beta }}_{o}\leq \sigma_{\max}$$

Minimization problem (22) is next placed into standard form [18] by making the substitution:

(25) $$\vec{y}={\tilde{R}}_{0}{\vec{\chi }}_{0}$$

The inverse of matrix ${\tilde{R}}_{0}$ now preprocesses the model matrix ${\tilde{T}}_{0}$, and, together with the new unknown $\vec{y}$, predicts the measured loss displacement contained in ${\vec{\delta }}_{l}$. Quadratic optimization problem (22) may be minimized by first decomposing the product, ${\tilde{T}}_{0}{\tilde{R}}_{0}^{-1}$, using singular value decomposition:

(26) $${\tilde{T}}_{0}{\tilde{R}}_{0}^{-1}=\tilde{U}\tilde{S}{\tilde{V}}^{T}$$

The measured loss displacement vector ${\vec{\delta }}_{l}$ is also processed, using the transpose of $\tilde{U}$ to create a modified loss displacement vector ${\vec{b}}^{\prime }$,

(27) $${\vec{b}}^{\prime }={\tilde{U}}^{T}{\vec{\delta }}_{l}$$

Defining a new unknown vector, $\vec{z}$, according to

(28) $$\vec{z}={\tilde{V}}^{T}\vec{y},$$

we end up with a new, but simpler constrained quadratic optimization problem:

(29) $$\min\;\;\;\;\frac{1}{2}{∥\tilde{S}\;\vec{z}-{\vec{b}}^{\prime }∥}^{2}+\frac{1}{2}\tau^{2}{∥\vec{z}∥}^{2}\;\;+\sum_{k=1}^{K}\lambda_{k}\sigma_{k}^{a}$$

From minimization problem (29), the relevant objective function can be written out in full as

(30) $$\begin{array}{c} L\left(\vec{z},\vec{\lambda }\right)=\frac{1}{2}\sum_{i=1}^{r}{\left(s_{i}z_{i}-{b^{\prime }}_{i}\;\right)}^{2}+\frac{1}{2}\sum_{i=r+1}^{M}{\left({b^{\prime }}_{i}\right)}^{2}+\frac{1}{2}\tau^{2}\sum_{i=1}^{N}z_{i}^{2}+\sum_{k=1}^{K}\lambda_{k}\sigma_{l(k)}^{a} \end{array}$$

As noted before, *N* is the number of mesh nodes, while *M* is the total number of inductive loss measurements available. Subscripts associated with unknowns in the Lagrange multiplier sum are meant to connect the indices of the two numbering schemes—one that tracks an unknown’s index number and a new index to track particular members of the current active set. If rank of the decomposed matrix in (26) equals *M*, then the second term in Equation (30) is absent.

To find the optimal solution, Equation (30) is minimized by setting each $\partial L/\partial z_{j}$ to zero, to give

(31) $$z_{j}=\frac{s_{j}{b^{\prime }}_{j}-\sum_{k=1}^{K}\lambda_{k}\Gamma_{l(k)j}}{s_{j}^{2}+\tau^{2}}\;\;\;;\;\;\;\;s_{j}=0\;\;\;\;\forall \;\;j>r$$

New composite matrix $\tilde{\Gamma }$ is defined as

(32) $$\tilde{\Gamma }=R_{o}^{-1}\tilde{V}$$

Also, setting $\partial L/\partial \lambda_{k}$ = 0 for each “*k*” in the active set gives an additional set of relations for members of the active set:

(33) $$\sigma_{l(q)}^{a}=\beta_{l(q)}+\sum_{j=1}^{N}\Gamma_{l(q)j}z_{j}=\sigma_{bq}^{a}\geq 0$$

Depending on the constraint applied to an active set member, $\sigma_{bq}^{a}$ is either the lower bound (=0) or the upper bound for member *q* in the active set. Combining Equations (31) and (33) to eliminate $z_{j}$ gives a relatively small set of linear equations for the Lagrange multipliers:

(34) $$\sum_{k=1}^{K}P_{qk}\lambda_{k}=\sum_{j=1}^{N}\Gamma_{l(q)j}z_{j}^{0}+\beta_{l(q)}-\sigma_{bq}^{a}\;\;\;\;;\;\;\;\;q=1,2,\ldots ,K$$

Matrix $\tilde{P}$ is defined by

(35) $$P_{qk}=\sum_{j=1}^{N}\frac{\Gamma_{l(q)j}\Gamma_{l(k)j}}{s_{j}^{2}+\tau^{2}}$$

Matrix P(q,k) is symmetric under interchange of indices *q* and *k*. Recall that l(q) and l(k) are intended to map a particular constrained variable *q* (or *k*) to its index “*l*” among all variables. The numerator of Equation (35) forms the dot product of row l(q) with row l(k), with each term adjusted by the jth denominator.

After solving for the Lagrange multipliers in Equation (34), Equation (31) is computed again, generally yielding a new active set, which calls for solving (34) again. The process is repeated until the active set is stable. All reconstructions reported here converge in fewer than ∼9 iterations.

### 2.3. Depth-Dependent Penalties

There are three levels of control over the penalties applied during image reconstruction. First, the global penalty parameter τ is sequentially set to progressively smaller values, chosen from the set of singular values obtained from the decomposition of Equation (26), though any value may be assigned. A reduction in τ reduces the penalty of each type, but is only reduced to the point where the computed error (first term of Equation (19)) becomes equal to the measured noise floor of the measurement system. Noise floor is obtained from a placebo scan without any phantom present on the 3D gantry stage, ∼0.9 mΩ. As τ approaches infinity, the solution not only approaches the average conductivity value, but also becomes smooth. As τ approaches zero, error falls below the known noise level in violation of the discrepancy principle so that spurious solutions are produced due to overfitting. Hence, determining the noise floor is important.

Secondly, specifying the ratio α/τ sets the relative magnitude of the two penalties. Setting this ratio to one permits each penalty to have nearly equal importance. If α/τ is set to far less than one, solution smoothness alone is emphasized without regard to the default solution. On the other hand, ratios much greater than one suppress departures from the default (average) solution without regard for smoothness.

Finally, either of the two penalties can be reduced with greater depth into the target, producing benefits similar to efforts used in diffuse optical tomography to restore interior sensitivity [19]. The rationale for penalty adjustment, in general, is to compensate for the much higher kernel values found nearer to coil windings. Locations where the kernel is larger are more strongly favored under image reconstruction. Penalty reduction is an effort to reduce, if not eliminate, the “unfair” emphasis given to locations where kernel values are persistently large. There are many choices available for adjusting a penalty to make it depth-dependent, though only one choice is presented here. Control of depth-dependence is feasible through either diagonal matrix $\tilde{D}$, or gradient matrix $\tilde{G}$ or both. A straightforward way to reduce the penalty for locations at greater depth is to use the kernel itself, as given by Equation (4). As an example, components of the diagonal matrix $\tilde{D}$ can be modified according to a scaled kernel:

(36) $$d_{j}=K\left(\rho ,\eta \;\left(z_{j}+z_{s}\right)\right)/K\left(\rho ,\eta z_{s}\right)$$

The radial distance ρ is usually chosen as the value that produces the maximum kernel value along the ρ axis for fixed *z* (see [4] for different coil types), while $z_{s}$ is chosen as some nominal average coil–target–boundary separation distance or possibly the closest approach distance. If sampling is confined to a single plane above the target, then the distance to that plane could be chosen as $z_{s}$. Here, $z_{s}$ was set to 2.0 mm for all calculations. The impact of other values for $z_{s}$ was not explored. Parameter η is commonly <1, with smaller values lessening the role of depth-dependency for a penalty.

Penalties could also be altered for locations falling outside of the scanning region in order to improve chances for target structure resolution in undersampled locations. A choice of lateral penalty dependence depends on whether the intention is to force remote locations toward a target average (increased penalty) or to increase sensitivity (decreased penalty) to structure outside the scanning region. As discussed in later sections, some phantom locations will fall outside of the *X* or *Y* scanning space, but no lateral alteration of penalty is used for this work. The expected consequence is that conductivity will tend toward the default solution outside the scanning region.

## 3. Materials and Methods

### 3.1. Phantom Construction and Properties

A single, low-conductivity phantom was constructed and scanned to provide a test for the dual-penalty image reconstruction algorithm. Scans were accomplished on a repurposed 3D printer, as discussed in the next section. The phantom was built up inside a plastic tray having internal dimensions of 29.8 × 29.8 cm square and 4.5 cm deep. An assortment of very-low-conductivity features was prepared, each consisting of an epoxy–wood–flour composite. Sufficient wood flour was added to a marine epoxy to yield a thick, but pourable, consistency. Wood flour also helped to promote an increase in both relative permittivity and conductivity. The composite was poured into forms of various sizes and shapes and was allowed to cure. After it was fully cured, electrical conductivity at ∼10 MHz was measured to be ∼0.1 S/m while relative permittivity was ∼8.

Four of the low-conductivity features were square blocks of dimension 4.0 × 4.0 cm (height) and 3.0 cm thick. Then, 2.2 cm diameter holes were drilled through two of these, in the horizontal thickness direction, while 1.9 cm holes were drilled through the remaining two blocks. Another set of four epoxy-based “fat” rectangular blocks had dimensions of 2.1 × 2.1 × 10.0 cm, while four additional “thin” rectangular blocks had dimensions of 1.5 × 1.5 × 10.0 cm.

**Figure 1:**
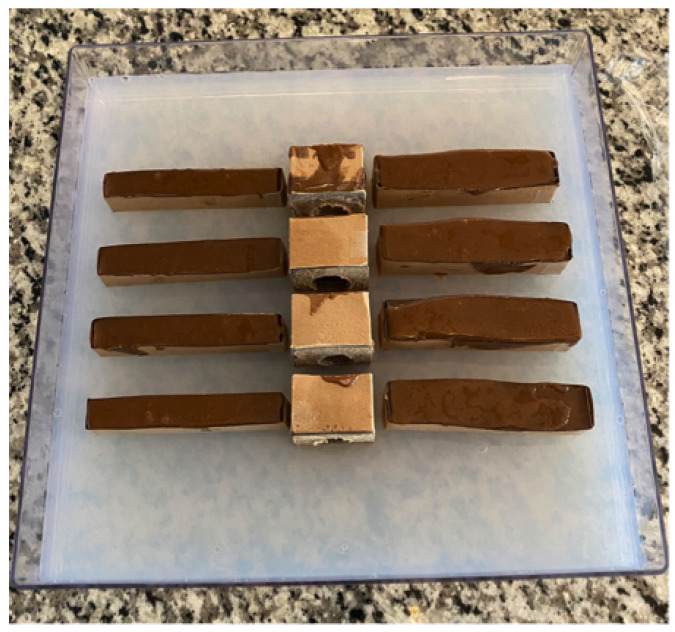
Layout of epoxy-based features within the supporting tray prior to complete filling with an agarose gel—the tray is partially filled with gel, as shown, to elevate the rectangular blocks to the mid-height of the tray. The central square blocks extend from the tray bottom to ∼4.0 cm of height above the tray floor. Note the holes drilled through the central square blocks.

The set of 4 square blocks were positioned parallel to each other, with holes coaxially aligned parallel to the *X*-axis of the tray and gantry, with 1.2 cm of spacing between adjacent pairs. The four fat rectangular blocks were positioned on one side of the row of centrally located square blocks, parallel to each other and the tray *Y*-axis. The thin rectangular blocks were also positioned parallel to each other and the tray *Y*-axis, but on the opposite side of the row of square blocks. Spacing between fat rectangular blocks was ∼1.5 to 2.0 cm while that between the thin rectangular blocks was ∼2.0 to 2.5 cm. The layout is shown in Figure 1, with the tray already partially filled with agarose gel to ensure that the eight rectangular blocks were elevated up to the vertical midpoint of the tray. The square blocks, however, touched the tray bottom and extended to a height of ∼4.0 cm. They were slightly buried by ∼0.2 cm of agarose.

**Figure 2:**
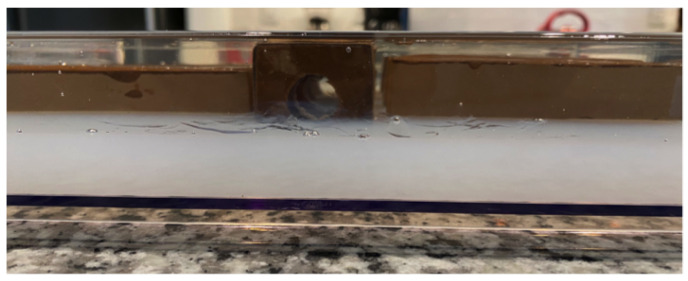
Edge view of phantom, immediately after pouring sufficient molten agarose to fill the upper half of the phantom, fill holes, and fully cover all low-conductivity features; the rectangular features are shown resting on the lower (white) portion of previously poured and cured agarose.

After placing the rectangular blocks into position, and allowing them to rest on the lower layer of previously cured agarose, additional doped agarose was poured into the tray to fully submerge all low-conductivity features. All agarose was doped with sufficient sodium chloride [20] to give a conductivity of ∼1.4 S/m at room temperature when cured. Sufficient agarose was poured into the tray so that the middle blocks were covered by ∼2 mm of gel, giving a total gel height of ∼4.3 cm, filling all holes. An edge view of the completely filled phantom, prior to gel solidification in the upper portion of the phantom, is shown in Figure 2. The key features of this phantom that challenge image reconstruction include the dimensions and locations of blocks, the gaps between blocks, and the holes through the central square blocks. Positioning the rectangular blocks out beyond the scanning region provides an additional challenge to image reconstruction.

### 3.2. 3D Scanning Gantry

Recent single-coil scans were accomplished manually, all while optically tracking coil position with an IR camera [21]. Though able to track the *X*, *Y*, and *Z* positions of the coil center to within ±0.25 mm each, together with coil orientation, the random nature of manual coil repositioning led to considerable sampling redundancy. Thus, a more methodical way of coil positioning is needed, but without sacrificing positioning accuracy. Hence, a 3D scanning gantry was used here that not only provides full control of sampling locations, but further improves upon coil positioning accuracy.

A discarded Creality Ender 3D printer was acquired and repurposed for the single-coil scanning measurements needed for image reconstruction. The print head was removed and modified to allow for mounting of the enclosure and its attached sensing coil. A custom stepper motor controller was built, and associated software was written to control the movement of each stepper motor mounted on the printer while simultaneously measuring inductive loss [13] at ∼8.85 MHz.

Stepper motors controlling movement along *X* and *Y* axes were configured to permit 0.0125 mm steps, while vertical steps were set to 0.01 mm. A 3D lattice of points was provided from a text file to direct the coil to desired locations where inductive loss in the sensing coil was measured [12,13] before moving on to the next location. The sensing coil consisted of four circular, parallel PCB traces, connected in series. Loop radii were 25.0 mm and coaxially spaced 0.3 mm apart to form a very short solenoid. Each trace was 0.5 mm wide, prepared from 2 oz. Cu, yielding a coil inductance of ∼2.35 μH for the complete coil.

**Figure 3:**
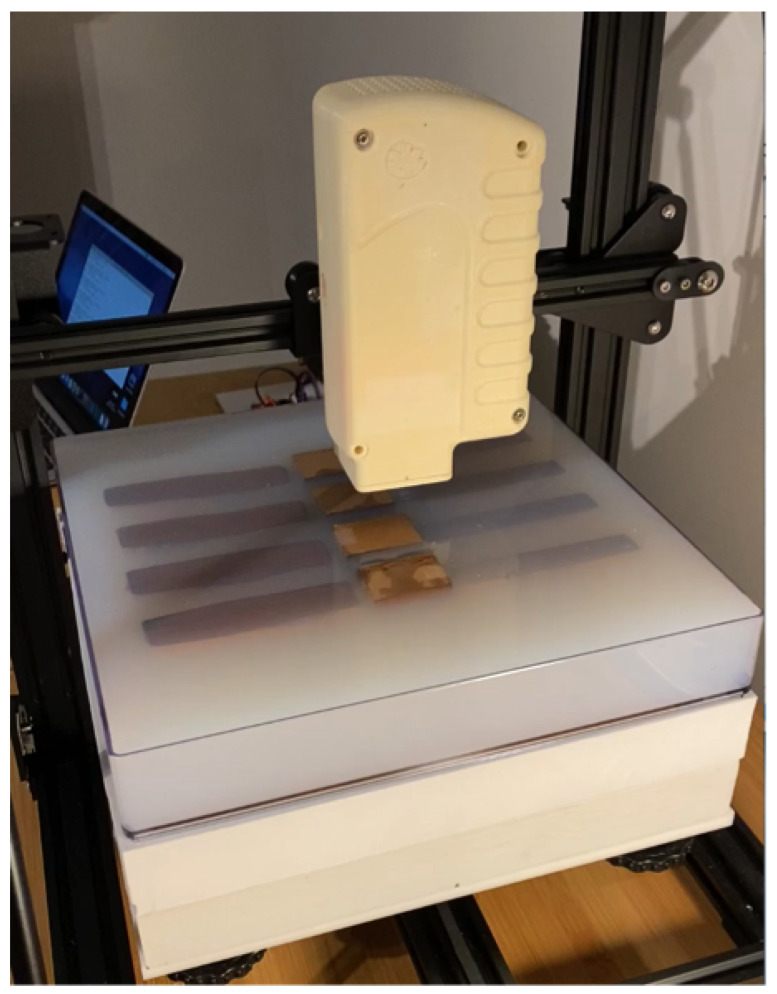
Discarded 3D printer repurposed for single-coil scanning experiments. The *Y*-axis runs from left to right tray edge, parallel to the embedded rectangular features, while the *X*-axis runs from the rear of the tray to the foreground tray border.

Two 31 × 31 cm × 4 cm thick EVA (ethylene vinyl-acetate co-polymer) foam slabs were stacked on the gantry stage, with the phantom then placed on top of the upper EVA slab. The purpose of the EVA is to provide some isolation from the metallic components comprising the gantry stage. The entire setup, with phantom in position, is shown in Figure 3.

A wide variety of scanning lattices is feasible with the setup shown in Figure 3, though only one is considered here. The primary constraints are the gantry support rods of the structure, which limits scanning along the *X* and *Y* borders. Referring to Figure 3, coordinate (0, 0, 0) mm is located at the rear left lower corner of the tray, while (298, 298, 0) mm is located at the front right lower corner of the tray. The particular lattice of sampling points featured in this work consists of 7 interleaved horizons, starting at *Z* = 45 mm and ending at *Z* = 57 mm. All horizons use a grid spacing of 13.5 mm. Odd numbered horizons run from (60.0, 60.0) mm to (249.0, 249.0) mm, while even numbered horizons run from (53.25, 53.25) mm to (242.25, 242.25) mm. All together, there are 1575 sampling locations across the lattice. An admittance measurement is acquired at (152.4, 152.4, 165) mm to establish a reference value, from which all loss values are computed [12,13]. Loss values are also corrected for a small amount of tank circuit loss due to parasitic capacitance [12]. Total scan time for this lattice is ∼10 min, though motors are easily programmed for faster scans.

**Figure 4:**
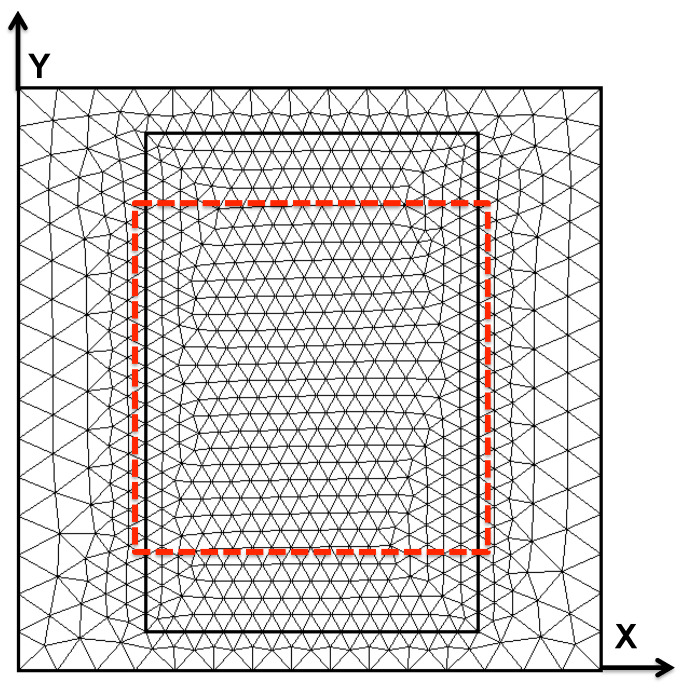
Finite element mesh extruded to form the six layers of prismatic elements. The inner black, bolded line is placed for mesh refinement over the region where low-conductivity features are located, while the dashed red line shows the sampling region. Rectangular blocks run parallel to the Y-axis.

To facilitate image reconstruction, the space occupied by the phantom was discretized into a finite element mesh consisting of six equally thick layers of prismatic elements spanning a total height of 4.3 cm. The 2D triangular mesh extruded to create the six layers is shown in Figure 4. The region delineated by the interior, bold rectangle contains all 12 low conductivity features previously described. The dashed red line indicates the maximal lateral extent of sampling, so that the coil center is never placed outside this dashed line.

Image reconstruction requires a stopping condition, where the global regularization parameter τ must not be reduced any further. Here, the Morozov discrepancy principle is used [22], which states that the computed error must not be reduced below a known measurement error. A measurement error is determined for the gantry system used here by performing “placebo” scans that include just the empty tray. For the lattice just described, a loss error of 0.9 mΩ was obtained. In all reconstructed images reported in the next section, regularization parameter τ was reduced only to the point that error—the normed difference between predicted and measured loss values—was ∼1.0 mΩ.

## 4. Image Reconstruction Results

**Figure 5:**
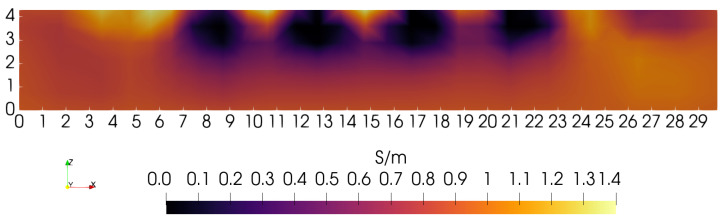
Sagittal slice at *Y* = 15 cm. The zero-order penalty is favored 10× over the first-order penalty. Conductivity at *Z*∼0 cm is near the average value of 0.94 S/m.

To illustrate the need for depth-dependent penalties, a sequence of “sagittal slices” (*Y* = 15 cm plane) is presented that does not use depth-dependence. The first sets the ratio α/τ = 10, so that the zeroth-order penalty term dominates over the first-order penalty. Figure 5 illustrates the *Y* = 15 cm sagittal slice obtained under this condition. Though the four low-conductivity blocks are showing at the correct position and width (∼3 cm), they do not show correctly over their complete height of ∼4 cm. The origin of this problem can be traced to the rapid decay of the kernel with depth, indicating a decreased sensitivity to material at greater depth. Of course, the coil cannot enter the phantom interior to compensate for such lost sensitivity. Fewer problems may be expected with the lateral resolution of features since the coil sensor can be moved in lateral directions with only modest restrictions. However, because of the limitations of lateral sensor movement on the 3D gantry used here, similar sensitivity problems are also expected that will hinder our ability to fully resolve the lateral extent of phantom features.

During the course of image reconstruction, the average electrical conductivity is computed as 0.94 S/m (via Equation (12) of [4]). Viewing the bottom of the image in Figure 5, conductivity is very close to this value. Even though the penalty is applied equally along the *Z*-axis, reconstruction suggests that the penalty is too high near Z=0, preventing any significant departure from the average near the lower boundary. The features that should be visible in the lowest portions of the image have collapsed to the average value. Apparently, the uniformly applied penalty is too large for these features to appear beneath ∼2.0 cm, though instrumentation noise likely also contributes to a lack of sensitivity to structure at depths exceeding ∼2 cm [4].

**Figure 6:**
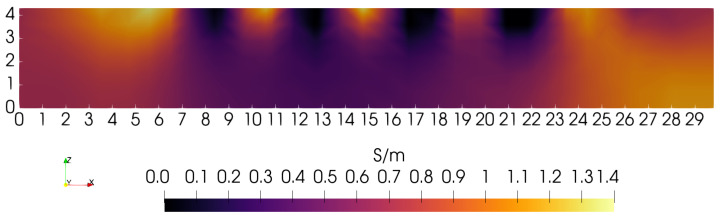
Sagittal slice, with the first-order normed penalty favored 10× over the zero-order penalty. Note the oversmoothed conductivity beneath ∼2 cm.

Examination of Figure 6 shows a similar behavior when the first-order penalty is strongly favored, by 10×. In this case, the lower portions of the square blocks emerge, but with excessive smoothing. Though conductivity is found to be well below the average value, the blocks are blurred together, giving a value of ∼0.35 S/m. This suggests that, again, the penalty imposed on solutions is excessive at greater depths into the phantom, limiting sensitivity to specific features.

**Figure 7:**
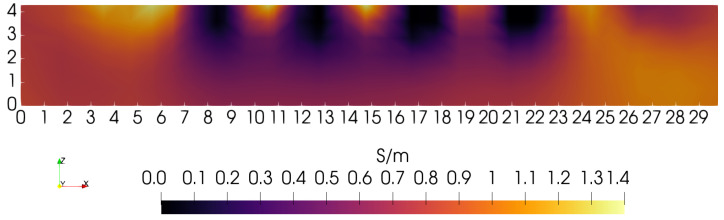
Sagittal slice obtained with each penalty given equal weight. Features are distinct as deep as *Z* = 2 cm.

When each penalty type is weighted equally, but still without depth-dependence, the sagittal slice shown in Figure 7 is obtained. In the absence of depth-dependent penalties, this is perhaps the best that can be achieved with this particular phantom, scanning lattice, and coil. In Figure 7, the blocks can be resolved up to a depth of nearly ∼2.5 cm. Note that this is the radius of the coil used for each of these three images. If coil radius is, indeed, a limiting factor for depth resolution when penalties are depth-independent, then a larger coil may give superior results.

Figure 5, Figure 6 and Figure 7 suggest that depth-dependent penalties may be helpful. Reducing the 0-order penalty at greater depths would allow conductivity to more easily deviate from its average value, while reducing the first-order penalty term at greater depths would prevent excessive smoothing of features as they appear. To reduce the complexity of managing two depth-dependent parameters, we first consider the simpler task that adjusts parameter $\eta_{0}$ (of Equation (36)) for the 0-order penalty, but in combination with the ratio α/τ. Increasing α/τ as the 0-order penalty is decreased at depth should limit smoothing as features appear.

**Figure 8:**
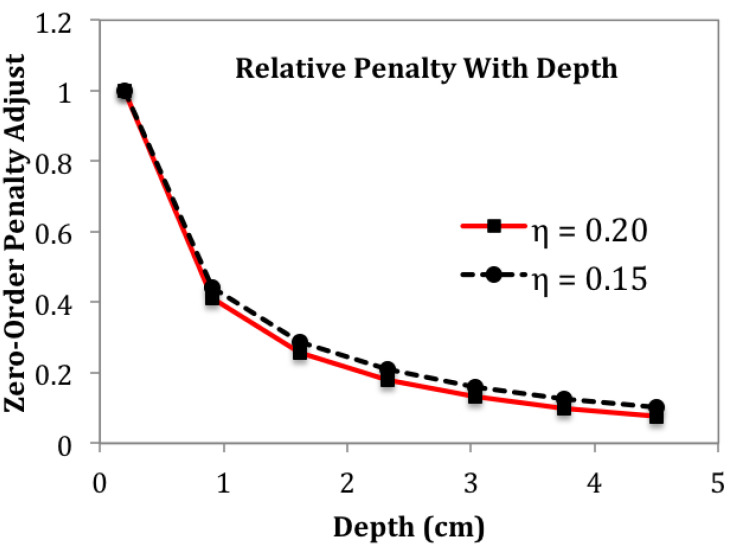
To improve sensitivity to features within the deeper layers of the phantom, the zero-order penalty is reduced with depth. Equation (36) is plotted for two values of the parameter η, to give a sense for its effect.

Setting $\eta_{0}$ of Equation (36) to 0.2, the reduction of the 0-order penalty term, with depth, is shown in Figure 8. The decrease shown in Figure 8 allows electrical conductivity to more easily deviate from the average during image reconstruction. To prevent excessive smoothing at greater depths when features emerge, as in Figure 6, α/τ is increased.

**Figure 9:**
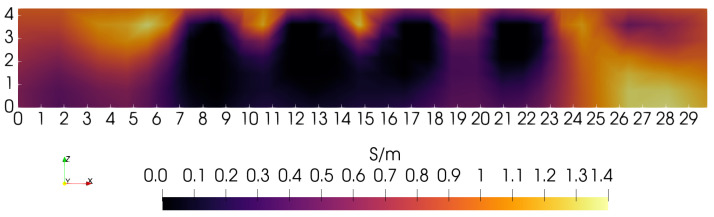
Sagittal slice at *Y* = 15 cm when the zero-order penalty is reduced according to the curve η = 0.2 shown in Figure 8 and α/τ = 10.

**Figure 10:**
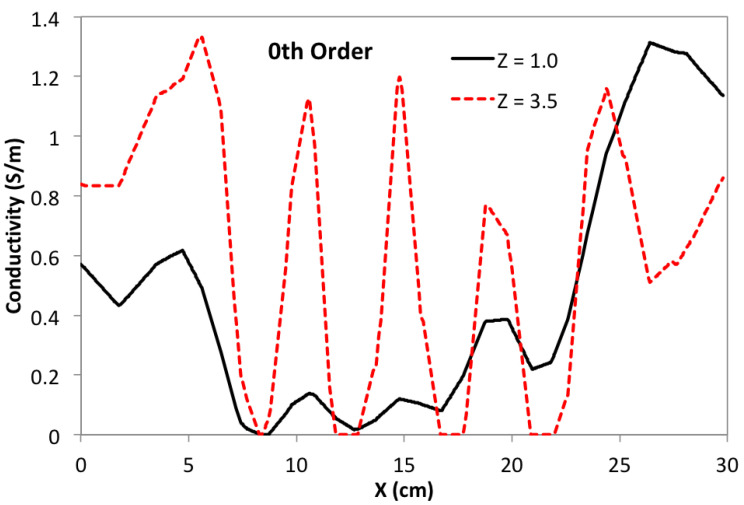
Line plot of conductivity at constant *Y* = 15 and *Z* = 1 cm or 3.5 cm. Note prominent peaks at *X* = 10.5, 15.0, and 19.5 cm at *Z* = 3.5 cm corresponding to gaps between features; near the bottom, at *Z* = 1.0 cm, the same gaps are only modestly noticeable.

Focusing again on the *Y* = 15 cm sagittal slice, and comparing with Figure 5, Figure 6 and Figure 7, Figure 9 shows the effects of using a depth-dependent zero-order penalty when α/τ = 10, the same as used in Figure 5. Setting this ratio to the same value as before helps to more clearly reveal the impact from assigning depth-dependence to the zero-order penalty. Making a direct comparison of Figure 9 with Figure 5 clearly shows that depth-dependence greatly improves the ability of the algorithm to reveal the square inclusions over their full depth. Though the square blocks are now plainly in view, we note that their separation is not as distinct near the bottom of the phantom as at the top. This can be more clearly demonstrated by building line plots across the image of Figure 9 exactly at *Y* = 15.0 cm and *Z* = 1.0 cm or 3.5 cm—shown in Figure 10. Note that the three gaps between blocks are very clearly discernible when *Z* = 3.5 cm, but are only modestly discernible at *Z* = 1.0 cm. Ideally, three identical square peaks should appear, having width ∼1.2 cm and heights reaching up to ∼1.4 S/m. Nevertheless, image reconstruction with depth-dependent penalties is clearly advantageous, improving sensitivity to features at greater depth.

**Figure 11:**
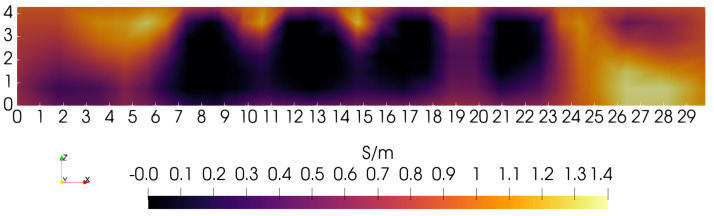
Sagittal slice at *Y* = 15 cm where both zero-order ($\eta_{o}=0.15$) and first-order ($\eta_{1}=0.10$) penalties are reduced with depth—see Figure 8; the ratio α/τ = 10.

**Figure 12:**
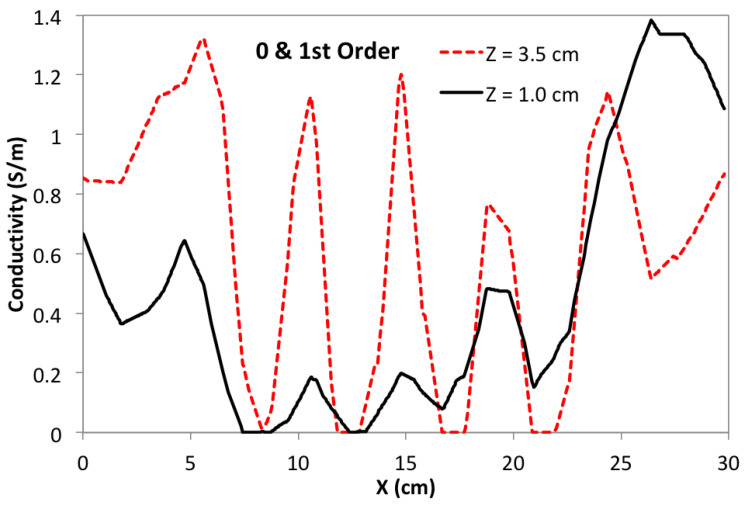
Line plot of conductivity at constant *Y* = 15 and *Z* = 1 cm or 3.5 cm, though now both penalty terms are reduced with depth into the phantom. Peaks located at *X* = 10.5, 15.0, and 19.5 cm for *Z* = 1.0 cm are somewhat more prominent than before.

To gain some sense of the impact of small changes in the 0-order penalty’s depth-dependence, another reconstruction was computed with $\eta_{0}$ reduced to 0.15, keeping α/τ = 10. The same sagittal slice showed no discernible change compared with Figure 9. In an effort to improve the image further, depth-dependence is also added to the first-order penalty. Although α/τ = 10, the first-order penalty still plays a significant role for locations Z<∼3.0 cm where the zeroth-order penalty has been reduced as much as 10× (see Figure 8). In fact, the two penalties are actually comparable in the lower half of the phantom. To explore the effect of adding depth-dependence to the first-order penalty, $\eta_{1}$ is set = 0.10 while $\eta_{0}$ remains = 0.15. The result is shown in Figure 11, and may be compared with Figure 9. The motivation for reducing the first-order penalty with depth is the appearance of excessive smoothing on the lower portions of the square feature at X= 21.75 cm in Figure 9. The sagittal slice for this case, as shown in Figure 11, indicates some further improvement in resolution, which is made clearer by building another line plot in the manner of Figure 10, which is shown in Figure 12. Note that the bottom portions of the two far right blocks are now somewhat more distinct, and are more filled out compared with the Figure 9 image.

Figure 9 and Figure 11 illustrate the benefits of reducing the size of the penalties with depth into the phantom for a sagittal slice. The spacing between reconstructed blocks is ∼1.0 cm while reconstructed block thickness is ∼3.0 cm—these results are close to actual physical dimensions. Importantly, the blocks are distinct over the full phantom depth of 4.3 cm, in spite of a coil radius that is only 2.5 cm. Similar results are found when smaller α/τ ratios are explored while adjusting depth-dependence on both zeroth- and first-order penalties, though no noticeable improvement in image fidelity was observed.

**Figure 13:**
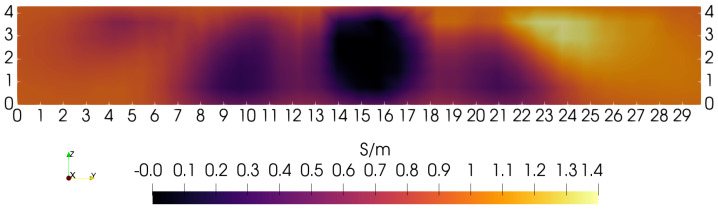
Transverse slice of the phantom, cutting through one of the large central square blocks, as well as the long rectangular blocks on either side. The central block is 4.0 × 4.0 cm, with a hole drilled into the center; the left block is 2.1 × 2.1 × 10 cm and the right block is 1.5 × 1.5 × 10 cm.

Figure 13 gives the *X* = 13.0 cm transverse slice for the same reconstruction as in Figure 11. The central block is resolved at the correct location, with the correct dimensions over its entire depth, and appears essentially square. However, the (1.9 cm diameter) hole is missing. Though the left-side rectangular block clearly appears to be larger than the right side block, as it should, neither block emerges over its full length. Truncation is likely due to insufficient sampling toward the edges of the phantom. Furthermore, vertical dimensions of both blocks are somewhat exaggerated due to smoothing.

**Figure 14:**
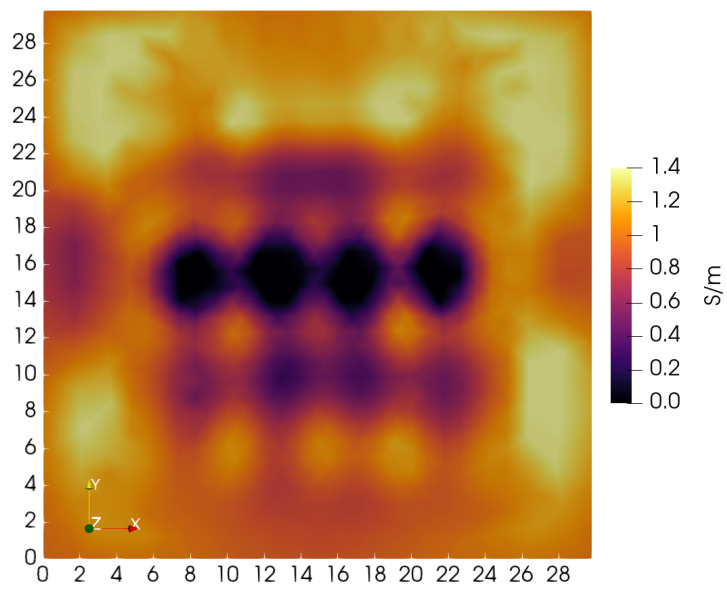
Tomographic slice taken of the XY plane at *Z* = 2.15 cm, which is located midway between top and bottom of the phantom. The four square blocks are clearly shown, but the lateral rectangular blocks are incomplete.

A mid-plane horizontal slice taken at *Z* = 2.15 cm is shown in Figure 14. This image also shows the failure of reconstruction to reveal the lateral rectangular blocks over their full length. Though the four square blocks fully appear and line up along the *X*-axis as they should, the conductivity is assigned a near-zero value, smaller than the expected 0.10 S/m. The depressed value may be a result of the sudden jump in relative permittivity, a feature not captured by the model Equation (1)—agarose gel relative permittivity is ∼72.

A gradual lateral reduction in penalty terms may help compensate for inadequate lateral sampling, possibly producing benefits comparable to the use of depth-dependence. Likely, the inadequacy of lateral sampling produces three unwanted results—central blocks show a depressed conductivity; side blocks are truncated; exaggerated side block vertical dimensions. Also, inadequate lateral sampling may have contributed to an absence of holes passing through the central square blocks. The depressed block conductivity suggests that the conductive holes might likewise be suppressed under reconstruction.

## 5. Discussion

As shown, combined zeroth- and first-order penalties work well when combined with depth-dependence, facilitating the resolution of central blocks over their full depth. This is in spite of a severely underdetermined image reconstruction problem, or using a coil with radius much smaller than the phantom’s depth. Future work with larger coils that are naturally able to promote greater target penetration may obviate the need for depth-dependent penalties. Nevertheless, the present work illustrates how the shortcomings of smaller coils may be addressed when larger coils are not an option. As Equation (1) indicates, larger coils, as well as higher frequency, improve sensitivity both nearer to and farther from coil windings. However, the discussion immediately preceding Equation (1) illustrates how the valid conductivity range is reduced with larger coils—doubling coil radius reduces the viable upper conductivity fourfold. An interesting approach, not yet tested, would be to merge data from larger and smaller coils, possibly improving image reconstruction throughout a target.

A similar adjustment of penalties along *X* and *Y* axes, to compensate for inadequate lateral sampling, was suggested as a means to fully resolve the rectangular features on either side of the row of centrally located square blocks. However, penalty reduction outside the XY region of these features, managed together with the manipulation of penalties over phantom depth, is likely to be a complex endeavor. A better approach would be to improve lateral scanning access, either by implementing a considerably larger 3D gantry or through the use of a flexible robotic arm, able to acquire data over larger distances and orientations. Depending upon phantom dimensions, results obtained thus far for single-coil MIT [4] suggest that lateral scans need to extend at least two coil diameters beyond a phantom’s edge, which is beyond the capability of the current 3D gantry.

## Data Availability

Code and data are not publicly available since they are proprietary property of Tayos Corp. However, both code and data can be made available after execution of a mutually acceptable nondisclosure agreement. The author may be contacted directly to arrange code and data sharing.

## Abbreviations

The following abbreviations are used in this manuscript:

MIT	Magnetic induction tomography
EVA	Ethylene vinyl acetate co-polymer
PCB	Printed circuit board
EM	Electromagnetic
RLC	Resistor–inductor–capacitance
